# Association Between Prevalence of Chronic Obstructive Pulmonary Disease and Health-Related Quality of Life, South Carolina, 2011

**DOI:** 10.5888/pcd10.130192

**Published:** 2013-12-26

**Authors:** Samuel Antwi, Susan E. Steck, Khosrow Heidari

**Affiliations:** Author Affiliations: Susan E. Steck, Arnold School of Public Health, University of South Carolina, Columbia South Carolina; Khosrow Heidari, Bureau of Community Health and Chronic Disease Prevention, South Carolina Department of Health & Environmental Control, Columbia, South Carolina.

## Abstract

**Introduction:**

We investigated the prevalence of chronic obstructive pulmonary disease (COPD) in various population subgroups in South Carolina and examined associations between COPD and 4 core measures of health-related quality of life (HRQOL).

**Methods:**

Data from 12,851 participants of the 2011 South Carolina Behavioral Risk Factor Surveillance System (BRFSS) were analyzed. COPD prevalence rates were age-adjusted to the 2000 standard US population. Logistic regression models were used to estimate adjusted odds ratios (AOR’s) and 95% confidence intervals (CIs).

**Results:**

The overall age-adjusted prevalence of self-reported diagnosis of COPD among community-dwelling adults in South Carolina in 2011 was 7.1% (standard error [SE] ±0.3). Prevalence of self-reported diagnosis of COPD was highest among women (8.9%; SE, ±0.5), those aged 65 years or older (12.9%; SE, ±0.5), current smokers (15.9%; SE, ±0.7), and those with low levels of education and income. Compared with community-dwelling adults without COPD, those with COPD were more likely to report fair or poor general health status (AOR, 3.97; 95% CI, 3.13–5.03), 14 or more physically unhealthy days (AOR, 2.10, 95% CI, 1.57–2.81), 14 or more mentally unhealthy days (AOR, 1.72; 95% CI, 1.21–2.43), and 14 or more days of activity limitation (AOR, 2.22; 95% CI, 1.53–3.22) within the previous 30 days.

**Conclusion:**

COPD is a highly prevalent disease in South Carolina, especially among older people and smokers, and it is associated with poor HRQOL. Future work aimed at reducing risk factors may decrease the disease prevalence, and increasing early detection and improving access to appropriate medical treatments can improve HRQOL for those living with COPD.

## Introduction

Chronic obstructive pulmonary disease (COPD) is a leading, yet under-recognized, cause of illness and death in the United States (1,2). COPD comprises 2 major lung diseases, chronic bronchitis and emphysema, and these 2 diseases often coexist (3,4). The best recognized symptoms of COPD are wheezing, shortness of breath, chronic cough, and chest tightness, which often occur as a result of airflow restriction induced by abnormal inflammatory response to inhaled noxious particles and gases such as tobacco smoke, occupational dust and chemicals, and fumes from biomass fuels (1–5).

Despite the well-established risk factors for COPD, few reliable, state-specific prevalence estimates across population subgroups exist (6–8). Similarly, state data have rarely been reported on the effect of COPD on health-related quality of life (HRQOL) comparing community-dwelling people with COPD with those without COPD (7,9). HRQOL is a major component of overall quality of life and an increasingly important outcome in the study of chronic diseases because it reflects a person’s physical and mental functional capacities and perceived general health status (10). It has been suggested that HRQOL should be incorporated into clinical practice guidelines for monitoring chronic disease severity and progression because it provides a personal and dynamic assessment of health status deterioration or improvement (11,12).

Although some sociodemographic differences in COPD prevalence have been reported for the Medicare population in South Carolina, these factors have not been studied in the entire adult population in the state (13). Similarly, no study has been conducted to examine the effect of COPD on HRQOL in South Carolina. To address this information gap, we conducted a large, population-based, cross-sectional study by using data from the Behavioral Risk Factor Surveillance System (BRFSS) to quantify the prevalence of COPD across various population subgroups in South Carolina, and we examined associations between self-reported COPD and HRQOL. Also, we examined how the HRQOL of people with self-reported COPD compares with the HRQOL of people with a self-reported history of myocardial infarction (MI) and people with a self-reported diagnosis of chronic kidney disease (CKD).

## Methods

### Data source and study subjects

This study used secondary data from the 2011 South Carolina BRFSS. The BRFSS is an ongoing, random-digit-dial telephone survey conducted by state health departments in partnership with the Centers for Disease Control and Prevention (CDC). Detailed description of the survey design and methodology is available elsewhere (14). BRFSS data are collected annually by using independent probability sampling of households. Trained interviewers make telephone calls to selected households to elicit information from civilian, noninstitutionalized adults aged 18 years or older on health, health risk behaviors, and health care access and use. In 2011, for the first time, the South Carolina BRFSS included a question on COPD, which provided the information needed to estimate COPD prevalence in the state. In 2011, the BRFSS program also introduced a raking system (iterative proportional fitting) that allows for incorporation of cellular phone users in the survey. This system ensures that previously underrepresented population subgroups are adequately captured in the survey (15). The overall weighted response rate for the South Carolina BRFSS in 2011 was 55% (landline, 60%; cellular phone, 43%), which compares favorably with the national response rate of 50% (landline, 53%; cellular phone, 28%) (16). The response rates were calculated on the basis of response rate formula number 4 of the American Association of Public Opinion Research (http://www.aapor.org/Standard_Definitions2.htm) (16). A total of 12,948 adults participated in the 2011 South Carolina BRFSS program. For the purposes of this study, we excluded respondents with missing data on COPD status (n = 97), leaving a total of 12,851 participants for final analyses. This study was reviewed by the institutional review board of the University of South Carolina and determined to be exempt.

### Study variables

COPD status was determined on the basis of survey participants’ response to the question, “Have you ever been told by a doctor or health professional that you have COPD, emphysema, or chronic bronchitis?” Affirmative response to this question was used to define the COPD study population. Survey participants who responded *no* to the question were used as the comparison group.

Differences in the prevalence of COPD were assessed by race, age, sex, education, income, smoking status, and health insurance coverage. Race was defined based on self-reported race/ethnicity and categorized as white, African American, or other. Because of the small number of survey participants representing racial/ethnic groups such as Hispanics, Native Americans, Alaskan Natives, Japanese, and Vietnamese, these groups were combined and classified as “other.” Age was categorized as 18 to 44 (referent group), 45 to 54, 55 to 64, and 65 or older. Educational level was based on the highest level of education completed by survey respondents and categorized as less than high school diploma or general equivalency degree (GED), high school diploma or GED, and college graduate (referent group). Information on income was based on respondents’ annual household income and reported by predetermined range categorized as less than $25,000, $25,000 to $49,999, and $50,000 or more (referent group). Smoking status was categorized as nonsmokers (referent group), former smokers, and current smokers. Data on smoking duration or dose (pack years) were not available. Health insurance coverage was reported as *yes* or *no* and used as such in the analyses.

HRQOL was measured by using the following health status and activity limitation measures: general health status, physical health, mental health, and impaired activity (hereafter referred to together as “unhealthy days measures”). General health status measures were based on survey participants’ subjective ratings of general health: “Would you say that in general your health is excellent, very good, good, fair, or poor?” General health was dichotomized into excellent-to-good, and fair or poor. Physical health was measured by the question: “Now, thinking about your physical health, which includes physical illness and injury, for how many days during the past 30 days was your physical health not good?” Mental health was measured by asking, “Now, thinking about your mental health, which includes stress, depression, and problems with emotions, for how many days during the past 30 days was your mental health not good?” To assess activity limitation, the study participants were asked, “During the past 30 days, for about how many days did poor physical or mental health keep you from doing your usual activities, such as self-care, work, or recreation?” Unfavorable HRQOL was defined as reporting fair or poor general health status rather than good or excellent, or an accumulation of 14 or more physically unhealthy, mentally unhealthy, and impaired activity days rather than fewer than 14 unhealthy or impaired activity days. These measures of unhealthy days are used in BRFSS data and have been validated as appropriate markers of substantial level of impaired HRQOL in the BRFSS population (10,17). In addition to examining HRQOL among respondents with self-reported diagnosis of COPD, we examined HRQOL for respondents with a self-reported history of MI and those with a self-reported diagnosis of CKD for comparison with other chronic diseases.

### Statistical analyses

The overall estimate of the COPD prevalence rate for South Carolina and COPD prevalence estimates across population subgroups were age-standardized to the 2000 US population (18), with the exception of those associated with specific age groups. We used a multivariate logistic regression model to estimate relative odds of COPD, adjusting for race, age, sex, education, income, smoking status, and health insurance coverage. Multivariate logistic regression models were also developed for each of the 4 unhealthy-days measures with adjustment for race, age, sex, education, income, smoking status, and health insurance coverage. These variables were included in each of the 4 logistic regression models to allow for comparisons between models and with findings of prior studies. All statistical analyses were performed using SAS version 9.3 (SAS Institute, Inc., Cary, North Carolina) with statistical significance set at α = 0.05 (2-tailed).

## Results

**Figure 1:**
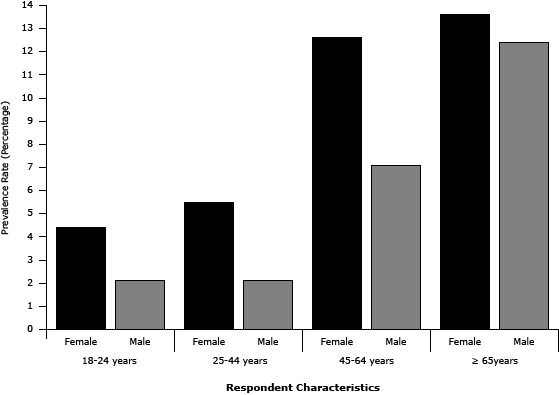
Prevalence of chronic obstructive pulmonary disease (COPD) by age group and sex. South Carolina, 2011. **Table:** 

The overall age-adjusted prevalence of self-reported COPD in South Carolina in 2011 was 7.1% (standard error [SE] ±0.3). This percentage equates to approximately 259,080 adults statewide. As expected, the prevalence of self-reported COPD increased with age, ranging from as low as 3.9% (SE, ±0.3) among those aged 18 to 44 years to as high as 12.9% (SE, ±0.5) among those 65 years and older. Women had higher prevalence of self-reported COPD (8.9%; SE, ±0.5) than did men (5.3%; SE, ±0.4). Self-reported diagnosis of COPD was consistently higher for women than for men across all age groups (Figure 1). The prevalence of self-reported COPD decreased significantly at higher levels of education and income. Respondents who had not attained a high school diploma or GED had higher prevalence of self-reported COPD (13.6%; SE, ≥1.2), than did those with a high school diploma or GED (6.9%; SE, ±0.4) and college graduates (3.3%; SE, ±0.4). Similarly, respondents reporting an annual household income less than $25,000 had higher prevalence of self-reported COPD (11.4%; SE, ±0.7) than did those with annual household incomes of $25,000 to $49,999 (6.1%; SE, ±0.7) and those with incomes of $50,000 or more (3.5%; SE, ±0.4). Self-reported COPD prevalence was highest among current smokers (15.9%; SE, ±0.7); former smokers also had higher prevalence of self-reported COPD (7.9%; SE, ±0.5) than did nonsmokers (3.6%; SE, ±0.2). However, self-reported COPD prevalence did not differ significantly by race or health insurance coverage. Results of the estimated odds ratios were similar to prevalence estimates (Table 1).

Respondent Characteristics		Prevalence Rate (SE)	95% CI
18–24 y	Female	4.4 (1.6)	1.2–7.6
	Male	2.1 (0.9)	1.3–3.9
25–44 y	Female	5.5 (0.8)	4.0–7.1
	Male	2.1 (0.4)	1.2–2.9
45–64 y	Female	12.6 (0.9)	10.7–14.4
	Male	7.1 (0.7)	5.6–8.6
≥65 y	Female	13.6 (1.0)	11.6–15.5
	Male	12.4 (1.1)	10.1–14.6

**Table 1:** Age-Adjusted Prevalence and Multivariate Adjusted Odds Ratios for Self-Reported Diagnosis of Chronic Obstructive Pulmonary Disease Among Adults Aged 18 years or Older, South Carolina Behavioral and Risk Factor Surveillance System, 2011

Characteristic	No. of Respondents^a^	No. with COPD	Age-Adjusted^b^ Prevalence, % (SE), 95% CI	Adjusted Odds Ratios, AOR^c^ (95% CI)
**Total (crude)**	12,851	1,216	7.6 (0.3), 6.9–8.2	NA
**Total (age-adjusted)**	12,851	1,216	7.1 (0.3), 6.5–7.8	
**Race**				
**White**	8,410	824	7.1 (0.4), 6.4–7.9	1.00 [Reference]
**African American**	3,166	264	6.3 (0.6), 5.3–7.4	0.69 (0.45–1.89)
**Other**	690	79	8.4 (1.6), 5.2–11.6	1.26 (0.68–2.36)
**Age Group, y**				
**18 - 44**	3,425	135	3.9 (0.3), 3.3–4.6	1.00 [Reference]
**45 - 54**	2,141	192	9.0 (0.6), 7.8–10.2	3.04 (2.16–4.27)
**55 - 64**	2,990	334	11.2 (0.6), 10.0–12.3	3.55 (2.57–4.81)
**≥65**	4,336	555	12.9 (0.5), 11.9–13.9	4.53 (3.29–6.24)
**Sex**				
**Male**	5,055	389	5.3 (0.4), 4.6–6.0	1.00 [Reference]
**Female**	7,796	827	8.9 (0.5), 7.9–9.9	1.91 (1.52–2.39)
**Education**				
**College graduate**	7,009	472	3.3 (0.37), 2.6–4.0	1.00 [Reference]
**High school diploma or GED**	4,037	433	6.9 (0.4), 6.6–8.7	1.41 (1.05–4.91)
**Less than high school or GED**	1,770	309	13.6 (1.2), 11.2–16.1	1.69 (1.17–2.42)
**Income**				
**≥$50,000**	3,765	173	3.5 (0.4), 2.6–4.4	1.00 [Reference]
**$25,000–$49,999**	2,966	242	6.1 (0.7), 4.7–7.5	1.59 (1.14–2.21)
**<$25,000**	4,106	592	11.4 (0.7), 10.1–12.8	2.89 (2.06–4.05)
**Smoking Status**				
**Nonsmoker**	6,703	309	3.6 (0.2), 3.2–4.1	1.00 [Reference]
**Former smoker**	3,715	484	7.9 (0.5), 6.9–8.9	2.67 (2.07–3.45)
**Current smoker**	2,350	417	15.9 (0.7), 14.5–17.4	4.58 (3.34–5.90)
**Health Insurance**				
**Yes**	10,846	1 037	6.9 (0.3), 5.2–8.7	1.00 [Reference]
**No**	1,948	176	9.0 (0.9), 7.4–10.6	1.10 (0.80–1.52)

Abbreviations: COPD, chronic obstructive pulmonary disease; SE, standard error; AOR, adjusted odds ratio; CI, confidence interval; NA, not applicable; GED, general educational development.

a Unweighted sample. Some categories may not sum to total survey sample because of missing data.

b Prevalence rates were age-standardized to the 2000 US population using the following age-groups: 18 to 24, 25 to 44, 45 to 64, 65 years or older, with the exception of age-group specific rates.

c Reflect adjustment for variables listed in the table with appropriate reference groups.

**Figure 2:**
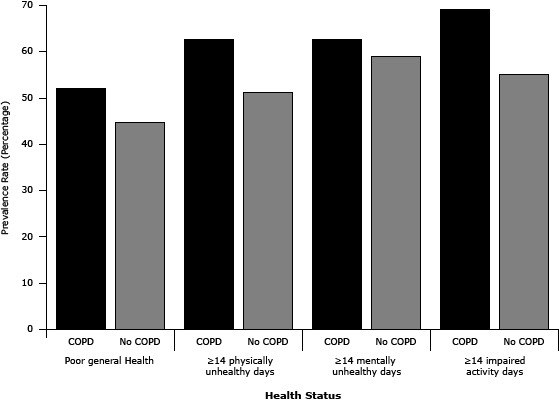
Age- and sex-adjusted prevalence of health-related quality of life by chronic obstructive pulmonary disease (COPD) status, South Carolina, 2011. **Table:** 

We examined associations between COPD and HRQOL by using multivariate logistic regression models with adjustment for sociodemographic variables (Table 2). Relative to those without COPD, survey respondents who reported a diagnosis of COPD were more likely to rate their general health status as fair or poor (adjusted odds ratio [AOR], 3.97; 95% CI, 3.14–5.03), and report 14 or more physically unhealthy days (AOR, 2.10; 95% CI, 1.57–2.81) in the previous 30 days. Similarly, respondents with self-reported COPD were more likely to report 14 or more mentally unhealthy days (AOR, 1.72; 95% CI, 1.21–2.43), and 14 or more days of activity limitation (AOR, 2.22; 95% CI, 1.53–3.22) in the previous 30 days compared with those without COPD. Respondents with COPD had poorer HRQOL across all unhealthy days measures (Figure 2).

**Table 2:** Adjusted Odds Ratios for Fair or Poor General Health Status, Physically Unhealthy Days, Mentally Unhealthy Days, and Impaired Activity During Previous 30 Days Among Adults With Self-Reported Chronic Obstructive Pulmonary Disease, History of Myocardial Infarction, or Chronic Kidney Disease, South Carolina, Behavioral Risk Factor Surveillance System, 2011

Measures of Health-Related Quality of Life		No. of Respondents^a^	COPD Status Yes/No					AOR^b^ (95% CI)	*P* Value		
**General Health** c											
**Chronic obstructive pulmonary disease**											
**Good-to-excellent**		9,793	482/9,311					1.00 [Reference]			
**Fair or poor**		2,980	719/2,261					3.97 (3.14– 5.03)		*P* < .001	
**History of myocardial infarction**											
**Good-to-excellent**		9,793	330/9,463					1.00 [Reference]		
**Fair or poor**		2,975	468/2,507					4.81(3.54–6.56)	*P* < .001	
**Chronic kidney disease**											
**Good-to-excellent**	9,818			154/9,664		1.00 [Reference]				
**Fair or poor**		2,979			267/2,712		6.22 (4.16–9.31)		*P* < .001	
**Physical Health**										
**Chronic obstructive pulmonary disease**											
**<14 unhealthy days**	2,472			258/2,214		1.00 [Reference]				
**≥14 unhealthy days**		1,946			511/1,435		2.10 (1.57–2.81)		*P* < .001	
**History of myocardial infarction**											
**<14 unhealthy days**	2,462			170/2,292		1.00[Reference]				
**≥14 unhealthy days**		1,961			286/1,675		1.50 (1.02–2.21)		*P* = .04	
**Chronic kidney disease**											
**<14 unhealthy days**	2,471			69/2,402		1.00 [Reference]				
**≥14 unhealthy days**		1,953			177/1,776		2.15 (1.23–3.77)		*P* = .007	
**Mental Health**											
**Chronic obstructive pulmonary disease**											
**<14 unhealthy days**	2,348			221/2,127		1.00 [Reference]				
**≥14 unhealthy days**		1,541			332/1,209		1.72 (1.21 - 2.43)		*P* = .002	
**History of myocardial infarction**											
**<14 unhealthy days**	2,359			116/2,243		1.00 [Reference]				
**≥14 unhealthy days**		1,544			147/1,397		1.14 (0.71 - 1.83)		*P* = .59	
**Chronic kidney disease**											
**<14 unhealthy days**	2,364			68/2,296		1.00 [Reference]				
**≥14 unhealthy days**		1,540			96/1444		1.70 (0.93–3.61)		*P* = .08	
**Impaired Activity**										
**Chronic obstructive pulmonary disease**											
**<14 unhealthy days**	1,495			187/1,308		1.00 [Reference]				
**≥14 unhealthy days**	1,312			372/940		2.22 (1.53–3.22)			*P* < .001	
**History of myocardial infarction**											
**<14 unhealthy days**	1,500			110/1,390		1.00 [Reference]				
**≥14 unhealthy days**	1,317			182/1,135		1.41 (0.86 – 2.33)			*P* = .17	
**Chronic kidney disease**											
**<14 unhealthy days**	1,497			55/1,442		1.00 [Reference]				
**≥14 unhealthy days**	1,325			110/1,215		2.47 (1.22 - 5.01)			*P* = .01	

Abbreviations: AOR, adjusted odds ratio; CI, confidence interval; COPD: chronic obstructive pulmonary disease.

a Unweighted sample. Some categories may not sum to total survey sample because of missing data.

b Odds ratios adjusted for race, age, sex, education income, smoking status, and health insurance coverage. Statistical analysis based on a 2-tailed test with (α = 0.05).

c Good-to-excellent include all those who indicated that their general health status is excellent, very good, or good.

Health Status		Prevalence Rate (SE)	95% CI
Poor general health	COPD	52.0 (3.4)	45.3–58.7
	No COPD	44.8 (4.6)	35.8–53.8
≥14 Physically unhealthy days	COPD	62.7 (4.5)	53.9–71.5
	No COPD	51.1 (6.7)	38.0–64.23
≥14 Mentally unhealthy days	COPD	62.6 (4.9)	53.0–72.2
	No COPD	59.0 (6.7)	45.9–72.1
≥14 Impaired activity days	COPD	69.2 (5.0)	59.7–79.3
	No COPD	55.2 (7.9)	39.7–70.7

HRQOL was also examined among respondents with a history of MI and those with a self-reported diagnosis of CKD and compared with HRQOL in the COPD study population. The odds of reporting fair or poor general health status was increased among those with self-reported COPD (AOR, 3.97; 95% CI, 3.14–5.03) and among those with a history of MI (AOR, 4.81; 95% CI, 3.54–6.56) and those with a self-reported diagnosis of CKD (AOR, 6.22; 95% CI, 4.16–9.31), when respondents with these diseases were compared with those without these diseases.

The odds of reporting 14 or more physically unhealthy days within the previous 30 days among those with self-reported COPD (AOR, 2.10; 95% CI, 1.57–2.81) were similar to those with a self-reported diagnosis of CKD (AOR, 2.15; 95% CI, 1.23–3.77) but higher than those with a history of MI (AOR, 1.50; 95% CI, 1.02–2.21), when respondents with these diseases were compared with those without these diseases. In terms of mental health, self-reported diagnosis of COPD was associated with significantly higher odds of reporting 14 or more mentally unhealthy days within the previous 30 days (AOR, 1.72; 95% CI, 1.21–2.43), but not self-reported history of MI (AOR, 1.14; 95% CI, 0.71–1.83) or self-reported diagnosis of CKD (AOR, 1.70; 95% CI, 0.93–3.61). The odds of reporting 14 or more days of activity limitation within the previous 30 days for those with self-reported COPD (AOR, 2.22; 95% CI, 1.53–3.22) was similar to the odds for those with a self-reported diagnosis of CKD (AOR, 2.47; 95% CI, 1.22–5.01); however, reporting a history of MI was not associated with activity limitation (AOR, 1.41; 95% CI, 0.86–2.33).

## Discussion

COPD is a growing, but often neglected public health problem. In 2010, a CDC workgroup embarked on a comprehensive evaluation of the state of knowledge on COPD. This process led to identification of several public health gaps, including limited public awareness of COPD prevalence and outcomes such as hospitalization rates, HRQOL, and mortality (19). This CDC workgroup identified COPD as an unmet public health need and proposed 4 primary goals to help meet this need, 1 of which is to increase public health research on COPD and prevention strategies (19). To help meet this goal, we investigated the prevalence of COPD across various population subgroups in South Carolina and examined associations between COPD and HRQOL. We observed a high prevalence of self-reported diagnoses of COPD in South Carolina with significant sociodemographic variations. Adults with self-reported COPD were more likely to report unfavorable HRQOL compared with those without COPD. The odds of a COPD patient reporting a fair or poor general health status were comparable with the odds of a person with a history of MI or a diagnosis of CKD. However, only a self-reported diagnosis of COPD was associated with poorer mental health in this study population.

The prevalence of self-reported COPD in South Carolina in 2011 was higher than the estimated 6.5% prevalence of self-reported COPD for the neighboring state of North Carolina and the national prevalence estimate of 6% (6). However, COPD prevalence estimates from self-reports often underrepresent the true prevalence of the disease in a population (8). Indeed, it has been demonstrated that a large number of people have undiagnosed COPD, which is primarily due to underuse of spirometry in assessing respiratory illness (20,21). For example, analyses of data from The Third National Health and Nutrition Examination Survey, which used information on spirometry diagnosis of COPD, suggest that over 50% of adults with evidence of COPD have never been given a diagnosis of the disease (5). This proportion is much higher for persons with mild-to-moderate disease severity (FEV_1_ <50% of predicted value) (20).

As seen in other populations (6–8,22–24), self-reported COPD in South Carolina was higher in older age groups and in women than in younger age groups and men. Although the higher prevalence of COPD in older age groups is not surprising because of the accumulation of risk factors over a lifetime, reasons why COPD is more prevalent among women are not well understood. However, sex differences in the physiological manifestations of the disease and in diagnostic patterns and susceptibility to the harmful effects of risk factors such as tobacco smoke have been noted (23). Accumulating evidence also suggests that COPD rates are higher in the poor and the least-educated populations than in the wealthy and well-educated populations (6,21,22,24). This finding is consistent with the COPD disparities observed in South Carolina. Of all the population subgroups examined, current smokers had the highest prevalence of self-reported COPD. This emphasizes the need for state-wide tobacco cessation policies and programs as a primary prevention measure. However, it is important to note that about a third of people with COPD never smoked (3,4). Therefore, researchers should explore preventive strategies targeting secondhand smoke exposure, occupational hazards such as exposures to noxious chemicals and dust, and exposure to environmental pollutants such as biomass fuel fumes.

This study also indicates that a self-reported diagnosis of COPD is associated with poor HRQOL. Those who reported a diagnosis of COPD had poorer HRQOL on all 4 core measures of HRQOL than did those without COPD. In particular, self-reported diagnosis of COPD was associated with a higher probability of unfavorable mental health; in contrast, self-reported history of MI and CKD were not associated with unfavorable mental health. This finding is especially noteworthy because it raises an interesting question about whether COPD imposes a greater burden in terms of affective disorders such as anxiety and depression than do other chronic diseases. This question warrants further investigation. It is reasonable to speculate that COPD plays a role in depression and anxiety through hypoxemia, a product of frequent dyspnea associated with the disease, which can trigger these affective disorders (25,26). It is also possible that these psychological states are precipitated by fear and misinterpretation of episodes of dyspnea and hyperventilation in COPD as more catastrophic events, escalating into psychological distress such as panic or generalized anxiety (27,28). The results, however, suggest that routine assessment of affective disorders, which can affect the disease course, should be incorporated into the clinical management of COPD.

The use of a large, population-based data that is representative of community-dwelling adults in South Carolina is one of the strengths of the study. Incorporation of cellular-phone-only households in the 2011 BRFSS survey also adds to the strength of the study, because it ensures that previously underrepresented population subgroups, particularly, low income, less educated, and younger populations who tend to maintain only cellular phones, are adequately captured in the survey. Inclusion of cellular-phone-only households in the BRFSS reduces nonresponse bias, ensures accurate estimation of population weights, and reduces error in statistical estimates (15).

The study findings are also subject to some limitations. First, the BRFSS data are based on self-report, which is subject to recall bias that may have resulted in either overestimation or underestimation of COPD prevalence rates. Second, BRFSS does not include COPD patients in long-term care facilities who may have substantial functional limitations and may have reported worse HRQOL than community-dwelling people with COPD. Additionally, this study did not examine COPD duration, severity, or treatment, which may contribute valuable information about HRQOL. Given the cross-sectional nature of the data, assessment of causal effect of COPD on HRQOL over time was not possible. Also, associations between COPD and HRQOL may be influenced by comorbidities. Adults with multiple chronic diseases generally have poorer quality of life than do those with few chronic conditions (29,30). It may have also been useful to examine COPD prevalence between cellular-phone-only and landline respondents. However, the iterative proportional fitting method (raking process) used in the statistical weighting process for the 2011 BRFSS data does not permit such analyses.

These results indicate that COPD is highly prevalent in South Carolina and is associated with relatively poor HRQOL. Targeted interventions aimed at reducing risk factors, such as smoking prevention and cessation policies and programs, may reduce the disease prevalence, and increasing early detection and improving access to appropriate medical treatments can improve HRQOL among those living with the disease.

## References

[1] Vestbo J , Hurd SS , Agusti AG , Jones PW , Vogelmeier C , Anzueto A , Global strategy for the diagnosis, management and prevention of chronic obstructive pulmonary disease, Gold Executive Summary. Am J Respir Crit Care Med 2013;187(4):347–65. 10.1164/rccm.201204-0596PP 22878278

[2] Halpern MT , Stanford RH , Borker R . The burden of COPD in the SA: results from the Confronting COPD Survey. Respir Med 2003;97 Suppl. C:S81–9. 10.1016/S0954-6111(03)80028-8 12647946

[3] National Institutes of Health, National Heart, Lung, and Blood Institute. Morbidity and mortality: 2012 chartbook on cardiovascular, lung, and blood diseases. Bethesda (MD): National Heart, Lung, and Blood Institute, 2012.

[4] American Thoracic Society/European Respiratory Society Task Force. Standards for the diagnosis and management of patients with COPD 2004 V. New York (NY): American Thoracic Society; 2004. http://www.thoracic.org/clinical/copd-guidelines/index.php. Accessed August 20, 2012.

[5] Mannino DM , Gagnon RC , Petty TL , Lydick E . Obstructive lung disease and low lung function in adults in the United States: data from the National Health and Nutrition Examination Survey, 1988–1994. Arch Intern Med 2000;160(11):1683–9. 10.1001/archinte.160.11.1683 10847262

[6] Centers for Disease Control and Prevention. Chronic obstructive pulmonary disease among adults — United States, 2011. MMWR Morb Mortal Wkly Rep 2012;61(46):938–43. 23169314

[7] Brown DW , Pleasants R , Ohar JA , Kraft M , Donohue JF , Mannino DM , Health related quality of life and chronic obstructive pulmonary disease in North Carolina. North N Am J Med Sci 2010;2(2):60–5. 22624116 10.4297/najms.2010.260 PMC3354436

[8] Mannino DM . COPD: Epidemiology, prevalence, morbidity and mortality, and disease heterogeneity. Chest 2002;121(5, Suppl):121S–6S. 10.1378/chest.121.5_suppl.121S 12010839

[9] Jackson BE , Suzuki S , Coultas D , Singh KP , Bae S . chronic obstructive pulmonary disease and health-related quality of life in the 2009 Texas Behavioral Risk Factor Survey. Health Educ Behav 2013; 40(4):469-79. 10.1177/1090198112460053 23041707

[10] Centers for Disease Control and Prevention. Measuring healthy days: population assessment of health-related quality of life. Atlanta (GA): Centers for Disease Control and Prevention; 2000. http://www.cdc.gov/hrqol/pdfs/mhd.pdf. Accessed August 20, 2012.

[11] Uzark K , King E , Spicer R , Beekman R , Kimball T , Varni JW . The clinical utility of health-related quality of life assessment in pediatric cardiology outpatient practice. Congenit Heart Dis 2013;8(3):211-8. 10.1111/chd.12002 22967147

[12] Harley C , Takeuchi E , Taylor S , Keding A , Absolom K , Brown J , A mixed methods approach to adapting health-related quality of life measures for use in routine oncology clinical practice. Qual Life Res 2012;21(3):389–403. 10.1007/s11136-011-9983-7 21822736

[13] Lopez-De Fede A , Mayfield-Smith K , Stewart J , Sudarshan N , Rodgers M , Sudduth D . Chronic obstructive pulmonary disorder (COPD) and SC Medicaid recipients: SFY 2010 Factsheet. Columbia (SC): Institute for Families in Society, University of South Carolina 2012. https://ifs.sc.edu/PRMM/Factsheets/2010/10COPD.pdf. Accessed August 20, 2012.

[14] Centers for Disease Control and Prevention. Behavioral Risk Factor Surveillance System: 2011 survey data. Atlanta (GA): US Department of Health and Human Services: Centers for Disease Control and Prevention; 2011. http://www.cdc.gov/brfss/annual_data/annual_2011.htm. Accessed August 20, 2013.

[15] Centers for Disease Control and Prevention. Methodologic changes in the Behavioral Risk Factor Surveillance System in 2011 and potential effects on prevalence estimates. MMWR Morb Mortal Wkly Rep 2012;61(22):410–3. 22672976

[16] Centers for Disease Control and Prevention. Behavioral Risk Factor Surveillance System, 2011 Summary Data Quality Report. Atlanta (GA): Centers for Disease Control and Prevention; 2013. http://www.cdc.gov/brfss/pdf/2011_Summary_Data_Quality_Report.pdf. Accessed August 25, 2012.

[17] Brown DW , Balluz LS , Heath GW , Moriarty DG , Ford ES , Giles WH , Associations between recommended levels of physical activity and health-related quality of life Findings from the 2001 Behavioral Risk Factor Surveillance System (BRFSS) survey. Prev Med 2003;37(5):520–8. 10.1016/S0091-7435(03)00179-8 14572437

[18] Klein RJ , Schoenborn CA . Age adjustment using the 2000 projected US population. Healthy People 2000; Stat Notes 2001:1-9. 11503781

[19] Centers for Disease Control and Prevention. Public health strategic framework for COPD prevention. Atlanta (GA): Centers for Disease Control and Prevention; 2011. http://www.cdc.gov/copd/pdfs/framework_for_copd_prevention.pdf. Accessed August 25, 2012.

[20] Mannino DM , Braman S . The epidemiology and economics of chronic obstructive pulmonary disease. Proc Am Thorac Soc 2007;4(7):502–6. 10.1513/pats.200701-001FM 17878461

[21] Mannino DM , Buist AS . Global burden of COPD: risk factors, prevalence, and future trends. Lancet 2007;370(9589):765–73. 10.1016/S0140-6736(07)61380-4 17765526

[22] Akinbami LJ , Liu X . Chronic obstructive pulmonary disease among adults aged 18 and over in the United States, 1998–2009. National Center for Health Statistics data brief no. 63. Hyattsville (MD): US Department of Health and Human Services, Centers for Disease Control and Prevention, National Center for Health Statistics; 2011. http://198.246.98.21/nchs/data/databriefs/db63.pdf. Accessed August 25, 2012.

[23] Han MK , Postma D , Mannino DM , Giardino ND , Buist S , Curtis JL , Gender and chronic obstructive pulmonary disease. Am J Respir Crit Care Med 2007;176(12):1179–84. 10.1164/rccm.200704-553CC 17673696 PMC2720110

[24] Buist AS , McBurnie MA , Vollmer WM , Gillespie S , Burney P , Mannino DM , International variation in the prevalence of COPD (The BOLD Study): a population based prevalence study. Lancet 2007;370(9589):741–50. 10.1016/S0140-6736(07)61377-4 17765523

[25] Maurer J , Rebbapragada V , Borson S , Goldstein R , Kunik ME , Yohannes AM , Anxiety and depression in COPD: current understanding, unanswered questions, and research needs. Chest 2008;134(4 Suppl):43S–56S. 10.1378/chest.08-0342 18842932 PMC2849676

[26] Cafarella PA , Effing TW , Usmani ZA , Frith PA . Treatments for anxiety and depression in patients with chronic obstructive pulmonary disease: a literature review. Respirology 2012;17(4):627–38. 10.1111/j.1440-1843.2012.02148.x 22309179

[27] Mikkelsen RL , Middelboe T , Pisinger C , Stage K . Anxiety and depression in patients with chronic obstructive pulmonary disease (COPD). A review. Nord J Psychiatry 2004;58(1):65–70. 10.1080/08039480310000824 14985157

[28] Laurin C . Moullec1 G, Bacon SL, Lavoie KL. Impact of anxiety and depression on chronic obstructive pulmonary disease exacerbation risk. Am J Respir Crit Care Med 2012;185(9):918–23. 10.1164/rccm.201105-0939PP 22246177

[29] Walker AE . Multiple chronic diseases and quality of life: patterns emerging from a large national sample, Australia. Chronic Illn 2007;3(3):202–18. 10.1177/1742395307081504 18083677

[30] Chen H-Y , Baumgardner DJ , Rice JP . Health-related quality of life among adults with multiple chronic conditions in the United States, Behavioral Risk Factor Surveillance System, 2007. Prev Chronic Dis 2011;8(1):A09 http://www.cdc.gov/pcd/issues/2011/jan/09_0234.htm . Accessed August 19, 2013. 21159221 PMC3044020

